# Unresolved Heart Block in Lyme Carditis: A Case Report

**DOI:** 10.7759/cureus.29661

**Published:** 2022-09-27

**Authors:** Shannon Baron, Subash Nepal, Madhab Lamichhane, Hal Roseman

**Affiliations:** 1 Cardiology, Upstate University Hospital, Syracuse, USA

**Keywords:** pacemaker, iv antibiotics, av conduction, complete heart block, lyme disease

## Abstract

A man in his thirties presented to the emergency department with a one-day history of syncopal episodes. He was found to have complete heart block and had multiple long and symptomatic pauses in telemetry while in the hospital. The longest pause was measured at 30 seconds. He had frequent occupational exposure to ticks and was found to have positive immunoglobulin G (IgG) and immunoglobulin M (IgM) antibodies for Lyme disease. He was immediately started on IV (intravenous) ceftriaxone and isoproterenol infusion for inotropy in anticipation of recovery of atrioventricular (AV) conduction with IV antibiotics. Rapid response was called for multiple symptomatic pauses overnight, the longest one lasting 30 seconds. The patient was taken for urgent temporary transvenous pacemaker placement in the morning. AV conduction failed to improve with IV antibiotics. A permanent pacemaker was placed on day four of hospitalization as his complete heart block failed to resolve with IV antibiotics and the patient could not be weaned from temporary pacemaker support.

A complete heart block is a rare manifestation of Lyme disease and warrants a high index of suspicion when a patient in an endemic area presents with this condition. A majority of patients recover with IV antibiotics, although some patients may need to be put on temporary pacemaker support in the interim. On rare occasions, a permanent pacemaker is necessary.

Atrioventricular conduction may fail to improve with IV antibiotics, and these patients may need early pacemaker support with a transvenous pacemaker in addition to IV ceftriaxone followed by permanent pacemaker placement. Our patient presented with recurrent Lyme disease and had a complete heart block on presentation, which failed to improve with IV antibiotics and required temporary transvenous pacemaker support followed by permanent pacemaker placement.

## Introduction

We are presenting a rare case of a man in his thirties who developed a complete heart block due to Lyme carditis, failed to improve with IV ceftriaxone, and required a permanent pacemaker. A complete heart block in Lyme disease generally resolves within a week with IV antibiotics and does not require a permanent pacemaker [1].

Lyme disease is caused by infection with the gram-negative spirochete bacteria, Borrelia burgdorferi, which is transmitted by the Ixodes tick. It is estimated that there are 476,000 cases of Lyme disease in the United States per year, making it the most common vector-borne disease [1]. It can cause a variety of manifestations, including an early erythema migrans rash and flu-like symptoms, which, if left untreated, can progress to cardiac, neurologic, or joint sequelae. The three stages of Lyme disease include the early localized, early disseminated, and late disseminated stages, with each occurring at different periods following initial infection. The early localized stage presents days to weeks following a tick bite, often manifesting as an erythema migrans rash with flu-like symptoms [2]. The early disseminated stage presents weeks to months after a tick bite with dermatologic, cardiac, and/or neurologic manifestations, while the late disseminated phase presents months to years later with neurologic and arthritic complaints [2].

Lyme carditis can occur in the early disseminated stage of Lyme disease and can present with symptoms including shortness of breath, chest pain, palpitations, dizziness, and syncope [3]. Diagnosis can be challenging when patients do not recall exposure to ticks or a recent tick bite. It is important to have a high clinical suspicion for Lyme disease in patients who live in an endemic zone and present with heart block.

## Case presentation

A man in his thirties presented to an emergency department (ED) with a one-day history of several episodes of syncope as well as dizziness and visual disturbances. The patient reported frequent exposure to ticks. Approximately two years ago, he was diagnosed with Lyme disease after presenting with an erythema migrans rash. He was treated with a two-week course of doxycycline. The patient reported a history of frequent tick bites.

**Figure 1:**
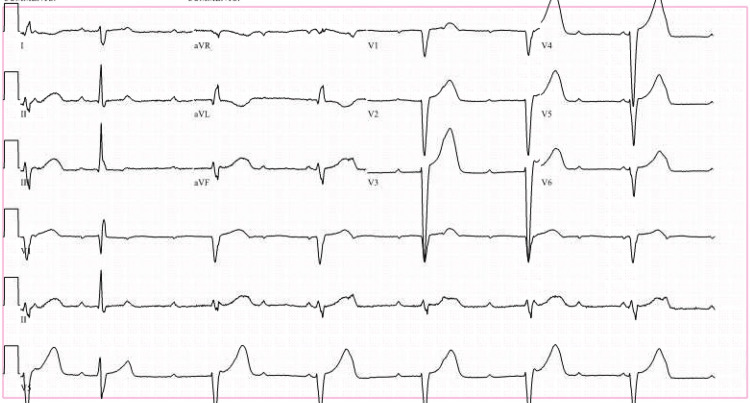
Initial ECG showing sinus rhythm with complete heart block and ventricular escape complexes

On presentation to the ED, his vital signs showed a blood pressure of 134/63, a pulse rate of 61/min, and a respiratory rate of 16/min, with O2 saturation of 99%. The echocardiogram (ECG) showed a complete heart block with a heart rate of 40/min and ventricular escape complexes (Figure 1). Lab work was remarkable for positive immunoglobulin G (IgG) and immunoglobulin M (IgM) antibodies for Lyme disease, an elevated white blood cell (WBC) count of 13,100, and a high Lyme C6 antigen titer (5.60 IV). He was admitted to the cardiac ICU for further management of a complete heart block due to Lyme carditis. He was started on an isoproterenol infusion and ceftriaxone 2 grams intravenously (IV) daily.

**Figure 2:**
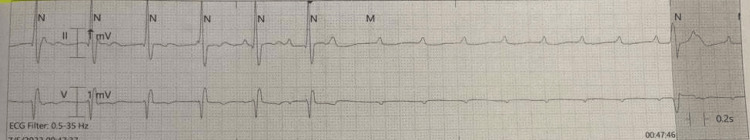
Telemetry strip showing a long ventricular pause

**Figure 3:**
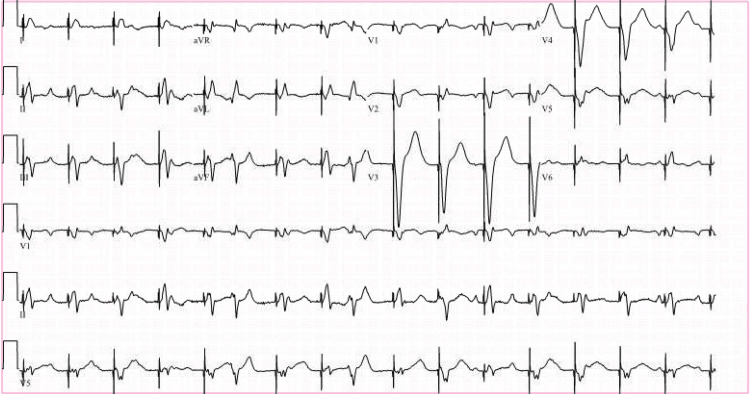
ECG showing sinus tachycardia with complete heart block and ventricular-paced rhythm while on temporary transvenous pacemaker

**Figure 4:**
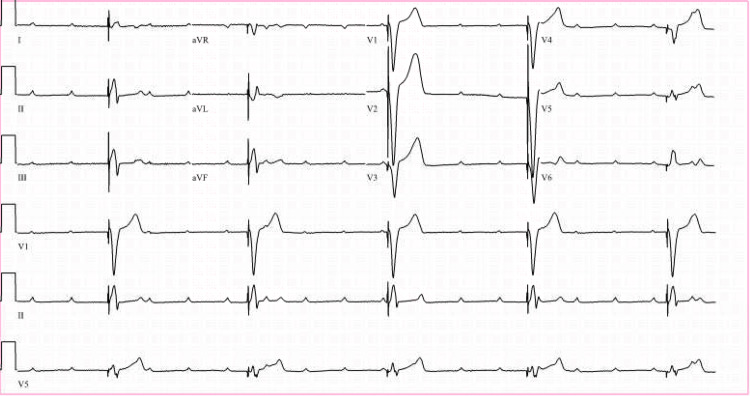
ECG showing sinus bradycardia with complete heart block with ventricular-paced rhythm at 30 bpm while temporary pacemaker support was lowered

On the night of admission, the patient had multiple symptomatic ventricular pauses lasting up to 30 seconds (Figure 2). A temporary transvenous pacemaker was emergently placed in the morning. The patient was put on a pacemaker backup rate of 50 bpm and required high energy of 15 milliamperes to capture (Figure 3). Weaning from pacemaker support was attempted but the patient went into complete heart block with ventricular-paced rhythm at 30 bpm and had a syncopal episode when the pacemaker rate was lowered (Figure 4). It was decided to place a permanent dual-chamber pacemaker as there was failed atrioventricular (AV) conduction at <90 bpm despite 72 hours of IV ceftriaxone while the patient was dependent on a temporary pacemaker. The patient was discharged on IV ceftriaxone therapy for a planned total of 21 days.

## Discussion

Lyme disease is caused by infection with spirochete bacteria of the genus Borrelia [4]. Borrelia (B.) burgdorferi is the species that causes nearly all cases of Lyme disease in the United States, while B. afzelii and B. garinii are seen in addition to B. burgdorferi in Europe and Asia [4, 5]. Ticks of the Ixodes genus can acquire and transmit infection with Borrelia species, with Ixodes (I.) scapularis being the most common vector in the United States [6]. The Ixodes tick has four life stages, including egg, larva, nymph, and adult, and they are most likely to transmit Lyme disease to humans during their nymph stage of life [6]. Humans are merely incidental hosts to the bacteria causing Lyme disease [6]. For transmission to occur, a tick must be attached to a human host for a minimum of 24 to 48 hours [7]. Most cases in the United States occur in the northeast region of the country, with Delaware, Connecticut, and Vermont having the highest incidence [5,6]. The geographic range of Lyme disease has expanded greatly in the last 30 years, along with a significant increase in incidence [6].

Lyme carditis is a rare manifestation of the early disseminated phase of Lyme disease and typically occurs in the first one to two months following initial infection [8]. It occurs in 0.3% to 4% of Lyme disease cases and most often presents as an atrioventricular block, with high-degree AV block occurring in 80% to 90% of patients with Lyme carditis [9]. Minor forms of AV block can rapidly progress to high-degree AV block within minutes, with the highest risk of progression in patients with a P-R interval >300 ms [2,9]. The pathophysiology of Lyme carditis is thought to be due to the infiltration of cardiac tissue by the infecting organism, leading to an immunologic response and cardiac injury [1]. The bacteria directly invade the connective tissue and cause lymphocyte-predominant inflammation [8]. There also may be an aspect of autoimmunity due to cross-reactivity between bacterial antigens and cardiac tissue, causing the IgM antibodies to attack the heart [1].

The prognosis for AV block related to Lyme disease is favorable when treated early with appropriate antibiotics. AV block typically resolves within the first ten days of antibiotic therapy [8]. If not diagnosed and treated early, Lyme carditis can require interventions such as temporary or permanent pacemaker placement. Temporary pacing is indicated for patients who present with symptomatic bradycardia, which encompasses about one-third of patients with Lyme carditis [8]. Placement of a permanent pacemaker is rarely needed and only recommended if there is failed conduction at <90 bpm, such as in our patient, or if there is no resolution of AV block by day 14 of antibiotic therapy [1]. An analysis of 103 Lyme carditis case reports showed the percentage of patients requiring a temporary pacemaker to be 35% and those requiring a permanent pacemaker to be 5.7% [2]. Only one of the patients with permanent pacemaker placement remained dependent on pacing [2]. Early recognition of Lyme carditis as the cause of heart block is crucial because it can typically be reversed with early antibiotic treatment, thus avoiding treatment with a permanent pacemaker [1].

## Conclusions

Lyme carditis is a rare but serious complication of Lyme disease which can affect the conduction system of the heart and cause complete heart block. Although patients with complete heart block due to Lyme carditis tend to recover with IV antibiotics, AV conduction may fail to recover and require permanent pacemaker placement. Lyme carditis should be suspected in a patient who presents with a complete heart block and is a resident of the Borrelia Burgdorferi endemic zone, like our patient. The patient may not recall a tick bite in the past. Early workup for Lyme disease with antibody-based tests and initiation of antibiotics is important. The threshold for the placement of a temporary pacemaker wire should be low in Lyme carditis complicated by complete heart block. People living in the endemic zone should be educated about taking precautions against tick bites and be advised to seek medical evaluation if they develop early clinical manifestations of Lyme disease, especially an erythema migrans rash.
